# Maturation of Corpus Callosum Anterior Midbody Is Associated with Neonatal Motor Function in Eight Preterm-Born Infants

**DOI:** 10.1155/2013/359532

**Published:** 2013-01-28

**Authors:** Preethi Mathew, Kerstin Pannek, Pamela Snow, M. Giulia D'Acunto, Andrea Guzzetta, Stephen E. Rose, Paul B. Colditz, Simon Finnigan

**Affiliations:** ^1^Perinatal Research Centre and UQ Centre for Clinical Research, The University of Queensland and Royal Brisbane and Women's Hospital, Brisbane, QLD 4029, Australia; ^2^School of Psychology and Psychiatry, Monash University, Melbourne, VIC 3806, Australia; ^3^Centre for Advanced Imaging, The University of Queensland, Brisbane, QLD 4027, Australia; ^4^Infant Neurology Section, Stella Maris Scientific Institute, 56018 Pisa, Italy; ^5^Grantley Stable Neonatal Unit, Royal Brisbane and Women's Hospital, Brisbane, QLD 4029, Australia

## Abstract

*Background*. The etiology of motor impairments in preterm infants is multifactorial and incompletely understood. Whether corpus callosum development is related to impaired motor function is unclear. Potential associations between motor-related measures and diffusion tensor imaging (DTI) of the corpus callosum in preterm infants were explored. *Methods*. Eight very preterm infants (gestational age of 28–32 weeks) underwent the Hammersmith neonatal neurological examination and DTI assessments at gestational age of 42 weeks. The total Hammersmith score and a motor-specific score (sum of Hammersmith motor subcategories) were calculated. Six corpus callosum regions of interest were defined on the mid-sagittal DTI slice—genu, rostral body, anterior midbody, posterior midbody, isthmus, and splenium. The fractional anisotropy (FA) and mean diffusivity (MD) of these regions were computed, and correlations between these and Hammersmith measures were sought. *Results*. Anterior midbody FA measures correlated positively with total Hammersmith (rho = 0.929, *P* = 0.001) and motor-specific scores (rho = 0.857, *P* = 0.007). Total Hammersmith scores also negatively correlated with anterior midbody MD measures (rho = −0.714, *P* = 0.047). *Discussion*. These results suggest the integrity of corpus callosum axons, particularly anterior midbody axons, is important in mediating neurological functions. Greater callosal maturation was associated with greater motor function. Corpus callosum DTI may prove to be a valuable screening or prognostic marker.

## 1. Introduction

Preterm infants are at a high risk of motor deficits in later life, with approximately fourteen percent of very preterm infants developing cerebral palsy (CP) [1], and up to forty percent of very preterm infants demonstrating mild motor deficits [2]. The mechanisms underlying such motor impairments have not yet been fully elucidated, but have been related to a number of factors, including abnormal cerebral development (particularly in sensorimotor regions) [3], conditions such as periventricular leukomalacia, peri-intraventricular hemorrhage [4], and/or stressors in the neonatal intensive care unit (NICU) environment [5]. New insights into the neural structures and mechanisms, underlying motor function in preterm-born infants, should help in the development of new diagnostic and prognostic tools and provide information on the immediate efficacy of early intervention therapies.

The corpus callosum is vital for communicating and integrating motor and somatosensory information between the hemispheres, and for bimanual motor coordination and function [6]. The maturation or structural development of the corpus callosum, that is, the organisation of axon fiber bundles and the degree of axonal myelination and microstructural integrity, can be studied using diffusion tensor imaging (DTI), particularly via the measures of fractional anisotropy (FA) and mean diffusivity (MD). The interpretation of FA and MD values is not entirely clear and largely depends on the underlying fiber architecture, including the degree of myelination, axon size, density, and organization [7, 8]. Since the corpus callosum is a highly organized myelinated structure, with axons typically running in packed parallel bundles, a high FA value and low MD value is suggestive of greater structural maturation [9]. In imaging studies, the infant corpus callosum is often segmented into six anatomical regions from anterior to posterior—the genu, rostral body, anterior midbody, posterior midbody, isthmus, and splenium [10].

Thompson et al. found that very preterm infants had significantly reduced corpus callosum cross-sectional area, lower FA and higher MD values compared to term infants, thus suggesting that corpus callosum development is altered in preterm-born infants compared to term-born infants [10]. A minority of studies have investigated the implications of this altered development by examining associations between corpus callosum maturation and neurodevelopment [11–13]. Rose et al. investigated the relationship between DTI measures of the genu and splenium (the most anterior and posterior subregions of the corpus callosum, resp.) taken at term age with neurological assessments (Amiel-Tison scale, gross motor function classification system, and Bayley scales of infant development) performed at eighteen months in preterm-born children [11]. The maturation of the splenium (as reflected by FA values) was significantly lower in children with abnormal versus normal neurological assessments, indicating that splenium FA near term age could potentially be used to prognosticate subsequent neurodevelopment [11]. These results are consistent with Hoon Jr. et al. 2002 study using DTI to examine white matter tracts in children with cerebral palsy as a result of periventricular leukomalacia. The tracts in the splenium, posterior corona radiate, and posterior internal capsule were markedly reduced in size in participants suffering from cerebral palsy compared to controls [14]. Hence, it was suggested that interhemispheric tracts to or from the sensory cortex, in addition to motor tracts, may also play a role in motor impairment [14]. Similarly, Rademaker et al. found a strong positive association between the midsagittal surface area of the corpus callosum on T1-weighted magnetic resonance imaging (MRI) scans and concomitant motor function in preterm-born children, studied at school age [15]. Furthermore, Iai et al. compared midsagittal corpus callosum T1-weighted MRI measures in preterm-born children with spastic diplegia (CP) versus neurologically typical children [16]. In the diplegic children, it was found that the ratios of the thickness of the splenium and of the midbody to the corpus callosum length were significantly reduced, compared to the ratios computed in neurologically typical children [16]. Furthermore, the ratio for the splenium was highly correlated with the extent of motor impairment [16]. This suggests that corpus callosum development is closely related to motor function.

However, aside from these few studies, potential associations between corpus callosum structure and neuro-motor function in preterm-born neonates have not been reported. Outcomes from such investigations may prove clinically meaningful; for example, they may inform development of early prognostic indicators, interventions, and/or markers to inform instigation of such interventions. Hence the primary aim of this study was to investigate and explore potential associations between DTI measures of subregions of the corpus callosum and concomitant neurological measures in a small sample of preterm infants. We hypothesized that a positive relationship would exist between corpus callosum maturation and concomitant neurological measures. 

## 2. Participants and Methods

### 2.1. Participants

The participants were preterm infants born at gestational age between 28 and 32 weeks (GA) at the Royal Brisbane and Women's Hospital (RBWH) with no post-natal medical issues or complications. Inclusion criteria were: infants that were born at a gestational age between 28 and 32 weeks, with a birth weight and length between the 10th and 90th percentiles for gestational age and who were determined to be medically stable. Exclusion criteria were as follows: the presence of abnormalities on brain ultrasound (i.e., intraventricular hemorrhage grades 3 or 4, persistent periventricular flares, or periventricular cysts) and the presence of major genetic disorders or malformations. These criteria were set to ensure that the study participants were at low risk for an adverse outcome and were medically stable. 

This study was approved by the RBWH Human Research Ethics Committee and Monash University Human Research Ethics Committee. Parents who agreed to their infant participation in the study signed a written consent form and were provided with a copy of the study protocol. 

### 2.2. Clinical Assessments

On reaching term-equivalent age (42 weeks GA), the infants underwent the Hammersmith neonatal neurological examination (HNE) [17] at the RBWH performed by a single neonatologist.

The HNE evaluates a number of motor and behavioral functions including posture and tone, tone patterns, reflexes, abnormal signs and orientation, and behavior [17]. When performed at around term age, it provides a summary score for each of these categories and a total HNE score (maximum score 32) which is the sum of these categories. As a follow-up analysis, a total “motor-specific” score (maximum 20) was calculated by summing the scores from the posture and tone, tone patterns, and reflexes categories. Associations between the HNE and motor-specific scores and DTI measures were examined. 

### 2.3. Neonatal Neuroimaging

The infants underwent neuroimaging at approximately term-equivalent age (refer to Table 1). The infants were scanned using a 3.0-T Siemens Tim Trio scanner and were placed in an MRI-compatible neonatal incubator with a dedicated head coil (Lammers Medical, Luebeck, Germany). The scans were performed during natural sleep after the infants had been fed. They were swaddled and placed in a vacuum fixation beanbag designed to keep the infant still and supported in the scanner. Diffusion weighted images were acquired along 30 directions at a *b*-value of 1000 s/mm^2^, along with one minimally diffusion weighted image (*b* = 0). The image resolution of the scans was 1.75 × 1.75 mm in-plane, with a slice thickness of 2 mm. Other imaging parameters were as follows: TR/TE 9300/130 ms, field of view 128 × 128, 47 slices. The acquisition time for the diffusion data was approximately five minutes. A field map was acquired to assist in the correction for susceptibility distortions. The MRI parameters for the field map acquired were TR/TE1/TE2 488/4.9/7.4 ms, matrix size 64 × 64, field of view 160 × 160 mm, slice thickness 2.6 mm with 0.65 mm slice gap, 29 slices, and flip angle 60 degrees. 

Preprocessing was performed using ANTS, FSL, and Freesurfer. Preprocessing of the imaging data involved correction for any head movement using rigid-body registration with subsequent adjustment of the *b*-matrix [18], skull stripping [19], as well as susceptibility distortion correction [20] and correction for intensity inhomogeneities [21]. Intensity outlier voxels caused by image artifact or head motion were detected and replaced [22]. Additionally, images were visually examined for artifacts and distortions and infants whose images showed motion artifact on the minimally diffusion-weighted image were excluded from the DTI analyses. 

**Figure 1:**
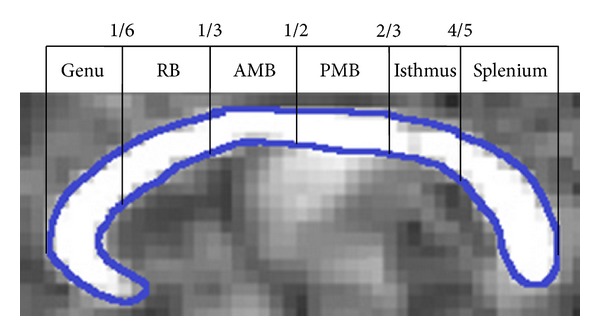
Corpus callosum segmentation schema adapted for neonates by Thompson et al. [10]. RB: rostral body; AMB: anterior midbody; PMB: posterior midbody.

Fractional anisotropy (FA), color FA, and MD maps of the brain were calculated for each infant, with FA representing the degree of anisotropic diffusion and MD representing the magnitude of diffusion. FA and MD were calculated using log-linear least squares with MRtrix [23]. Alignment of images with the midsagittal plane was achieved using rigid-body registration of FA maps with the John Hopkins University neonatal FA template. This transformation was then applied to the color FA and MD maps. Care was taken in the manual delineation of the corpus callosum in order to minimize the number of voxels that were contaminated by partial volume effects [24]. For each infant, based on visual inspection, a corpus callosum region of interest was manually drawn onto the midsagittal slice of the color-FA maps. Four sets of masks were drawn for each infant, to ensure reproducibility of the masks. For each infant, the four corpus callosum masks were then segmented into six regions—genu, rostral body, anterior midbody, posterior midbody, isthmus, and splenium—according to the neonatal segmentation schema provided by Thompson et al. (see Figure 1) [10]. The mean FA and MD were then determined for each region. An average was taken of the mean values of the regions from the four masks to provide six corpus callosum FA and MD values for each infant. 

### 2.4. Statistical Analyses

The infants ranged between 41 and 45 weeks GA at the time of DTI assessment. As it is known that FA values tend to increase rapidly with increasing GA [25], the FA values were adjusted for GA. Using SPSS (Statistical Package for the Social Sciences, version 14.0; IBM SPSS, Chicago, IL,USA), the correlations between clinical measures and corpus callosum DTI measures were explored using a two-tailed Spearman's rho (see Tables 2 and 3 for the FA and MD values used in the analysis). Correction for multiple comparisons was not performed given the exploratory nature of this study of a small sample as well as the number and nature of the variables analyzed. 

## 3. Results

Thirteen preterm infants were recruited into the study. Table 1 provides a summary of the characteristics and assessments for these thirteen infants. 

Due to time constraints, MRI was not performed for two infants. Furthermore, due to technical issues and motion artifacts, DTI data from three infants was excluded from the analyses, leaving eight infants whose data was included in the correlation analyses. 

### 3.1. Clinical and DTI Correlation Analyses

**Figure 2:**
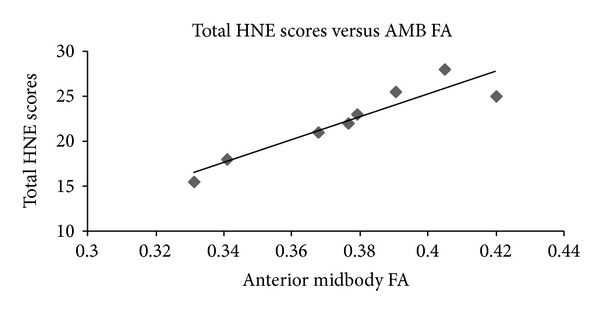
Scatterplot of total HNE scores versus anterior midbody FA (*n* = 8; spearman's rho = 0.929; *P* = 0.001).

**Figure 3:**
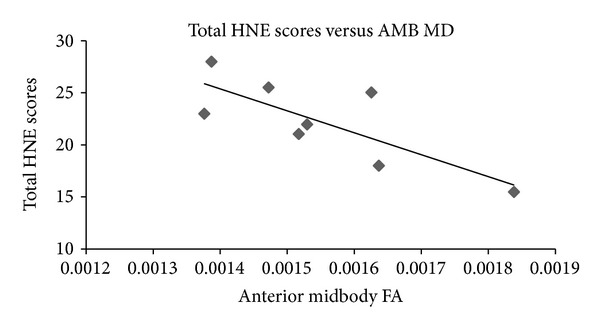
Scatterplot of total HNE scores versus anterior midbody MD (*n* = 8; spearman's rho = −0.714; *P* = 0.047).

**Figure 4:**
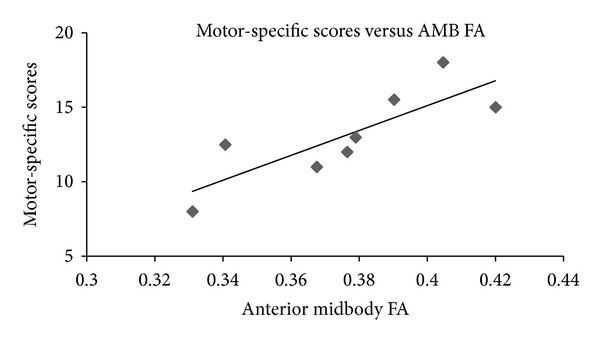
Scatterplot of motor-specific scores and anterior midbody FA (*n* = 8; spearman's rho = 0.857; *P* = 0.007).

Using Spearman's rho, associations were explored between the clinical and DTI measures (see Tables 4 and 5). A highly significant positive correlation was found between the total HNE scores and the anterior midbody FA measures (rho = 0.929; *P* = 0.001) and similarly between the motor-specific scores and the anterior midbody FA measures (rho = 0.857; *P* = 0.007). A statistically significant negative correlation was found between the total HNE scores and the anterior midbody MD measures (rho = −0.714; *P* = 0.047). Scatter plots illustrating these correlations are provided in Figures 2, 3, and 4. 

In summary, the total HNE scores correlated significantly with both the anterior midbody FA and MD measures. In addition a significant positive correlation was found between the motor-specific subscores of the HNE assessment and anterior midbody FA measures. 

## 4. Discussion

Overall neurological function, as well as specific motor function measures, correlated significantly with DTI measures from the anterior midbody, but not from other regions of the corpus callosum. These findings are consistent with studies investigating corpus callosum topography in primates and humans [26, 27]. The results from these studies suggest that, from a neuroanatomical perspective, axons in the midbody interconnect the premotor and primary motor cortical areas. Furthermore, the primary and secondary motor cortices and somatosensory regions are located in the precentral and postcentral cortices, which are in a similar spatial region to the corpus callosum midbody [28]. Given that these somatosensory and motor regions, as well as the corpus callosum, are topographically organized, it is likely that interhemispheric axons which connect homologous regions of the left and right cortical regions pass through the corpus callosum midbody. In particular, these study findings suggest that such axons connecting (frontal) motor cortical regions likely constitute part of the anterior midbody. 

Both total HNE and motor-specific scores correlated with greater maturation of the anterior midbody (i.e., higher FA and lower MD values). It has been suggested that FA values in the corpus callosum are primarily a reflection of fiber density and organization, rather than myelination or axon diameter [29]. Hence, higher FA values are suggestive of advanced callosal tract organization. In contrast, MD values are believed to be more sensitive to (pre-)myelination [30], with lower MD values indicating advanced callosal myelination and microstructure [30, 31]. Consequently, a relatively well-organized and (pre-)myelinated corpus callosum (e.g., anterior midbody) may be associated with more efficient transmission of interhemispheric neural signals, which thereby permits more optimal processing and integration of (e.g., motor-related) information between the hemispheres.

This study appears to be one of the first studies to describe a strong correlation between the maturation of the anterior midbody and concomitant neurological function in preterm infants. In contrast, a number of studies (as described in Section 4) demonstrated associations between splenium maturation and altered neurological function. However, the infant cohorts used in these studies involved infants with demonstrated white-matter injury and periventricular leukomalacia (PVL). A characteristic feature of PVL is thinning or altered maturation of the splenium; hence, this may explain why a strong association was found between splenium maturation and altered neurological function in these infants. 

In light of these findings, intervention programs and management strategies directed towards enhancing the development of white matter should be investigated further, as this may aid in enhancing motor function and possibly other neurobehavioral functions for preterm infants. An MRI study performed by Smith et al. demonstrated that stresses from the NICU environment correlated with decreased white matter maturation and interhemispheric communication in preterm infants, as assessed by DTI and functional MRI [5]. This study also reported that infants experiencing greater NICU-induced stress in the immediate postnatal period demonstrated poorer motor function at term-equivalent age [5]. Hence, NICU stressors may adversely influence white matter development and neurodevelopmental outcomes. Als et al. performed a randomized control trial investigating the merits of a Newborn Individualized Developmental Care and Assessment Program (NIDCAP) in order to reduce stressors for preterm infants while in NICU [32]. They found improved white matter development (assessed via DTI), brain functional connectivity (as measured by electroencephalography), and neurological function in the NIDCAP cohort of infants versus controls at term-equivalent age. A similar study performed by McAnulty et al. demonstrated that NIDCAP intervention had positive effects on preterm-born children's neurobehavioral function at least until school age [33]. Since the current findings indicate an association between anterior midbody development and neurological function, this may in part explain why interventions such as the NIDCAP, which are directed towards enhancing white matter development, resulted in improved neuromotor function.

The observed relationship between anterior midbody integrity and neurological function, if replicated and extended in larger studies, may inform future development of prognostic tools for neonates. Future studies should investigate whether such DTI measures may demonstrate value in the prediction of neurodevelopmental, particularly motor outcomes at a later age. Conversely, in light of the strong correlation between HNE scores and DTI measures, it is possible that HNE assessment may also prove to be a valuable prognostic marker, with the added advantage of being easy to perform at the bedside. Such markers might also ultimately prove informative for future interventional trial programs, for example for selection of inclusion criteria, or predicting or assessing response to a given intervention.

There are a number of limitations in the current study, particularly the small sample size and associated low statistical power [34]. In addition, the corpus callosum segmentation method employed was not necessarily optimal as current understanding of corpus callosum anatomical segmentation and tract topography in infants is limited, unlike in adult studies. This would be likely improved by identification of callosal regions defined by DTI tractography methods in infants [35]. However, this small study has delivered some novel findings which may inform future studies.

## 5. Conclusion

A positive association was observed between DTI measures of structural maturation of the anterior midbody of the corpus callosum and neurological and motor function, in healthy preterm-born infants assessed at term-equivalent age. Future studies of larger samples may extend upon these preliminary findings and possibly investigate the value of corpus callosum DTI measures as early prognostic markers for neurodevelopmental and motor outcomes.

## Figures and Tables

**Table 1:** Descriptive data pertaining to infant characteristics, clinical assessment scores, and GA at assessments.

	Mean	Range
GA at birth	31.2 weeks	28.6–32.6 weeks
Birth weight	1486.8 g	1064–1717 g
Total HNE score	22.3	15.5–28.0
Motor-specific score	12.9	8.0–18.0
GA at clinical assessment	42.2 weeks	41.1–44.6 weeks
GA at MRI	42.7 weeks	41.1–44.6 weeks

**Table 2:** Corpus callosum fractional anisotropy values for each infant.

	Genu	Rostral body	Anterior midbody	Posterior midbody	Isthmus	Splenium
Infant 1	0.35	0.29	0.34	0.46	0.32	0.39
Infant 2	0.35	0.37	0.40	0.33	0.32	0.49
Infant 3	0.36	0.32	0.38	0.30	0.32	0.39
Infant 4	0.38	0.34	0.39	0.31	0.29	0.41
Infant 5	0.35	0.34	0.38	0.42	0.33	0.44
Infant 6	0.35	0.37	0.42	0.49	0.47	0.48
Infant 7	0.38	0.37	0.37	0.36	0.37	0.45
Infant 8	0.36	0.34	0.33	0.35	0.42	0.44

**Table 3:** Corpus callosum mean diffusivity values for each infant.

	Genu	Rostral body	Anterior midbody	Posterior midbody	Isthmus	Splenium
Infant 1	0.0016	0.0018	0.0016	0.0014	0.0016	0.0015
Infant 2	0.0016	0.0014	0.0014	0.0015	0.0015	0.0015
Infant 3	0.0014	0.0016	0.0014	0.0016	0.0017	0.0017
Infant 4	0.0015	0.0015	0.0015	0.0014	0.0015	0.0015
Infant 5	0.0017	0.0016	0.0015	0.0013	0.0016	0.0014
Infant 6	0.0017	0.0017	0.0016	0.0014	0.0012	0.0015
Infant 7	0.0014	0.0015	0.0015	0.0014	0.0012	0.0012
Infant 8	0.0015	0.0017	0.0018	0.0017	0.0013	0.0013

**Table 4:** Correlation between clinical scores and corpus callosum FA measures.

	Fractional anisotropy					
	Genu	Rostral body	Anterior midbody	Posterior midbody	Isthmus	Splenium
Total HNE						
Rho	0.000	0.476	0.929**	−0.333	−0.476	0.381
*P *	1.000	0.233	0.001	0.420	0.233	0.352
Motor-specific						
Rho	−0.238	0.238	0.857**	−0.262	−0.548	0.214
*P *	0.570	0.570	0.007	0.531	0.160	0.610

**P < 0.01.

**Table 5:** Correlation between clinical scores and corpus callosum MD measures.

	Mean diffusivity					
	Genu	Rostral body	Anterior midbody	Posterior midbody	Isthmus	Splenium
Total HNE						
Rho	0.095	−0.643	−0.714*	0.000	0.048	0.357
*P *	0.823	0.086	0.047	1.000	0.911	0.385
Motor-specific						
Rho	0.190	−0.405	−0.595	0.119	0.214	0.595
*P *	0.651	0.320	0.120	0.779	0.610	0.120

*P < 0.05.

## Abbreviations

GA: Gestational age
CP: Cerebral palsy
NICU: Neonatal intensive care unit
NIDCAP: Newborn individualized developmental care and assessment program
DTI: Diffusion tensor imaging
FA: Fractional anisotropy
MD: Mean diffusivity
MRI: Magnetic resonance imaging
HNE: Hammersmith neonatal neurological examination.
